# Barriers and facilitators to physical activity amongst overweight and obese women in an Afro-Caribbean population: A qualitative study

**DOI:** 10.1186/s12966-015-0258-5

**Published:** 2015-07-28

**Authors:** Miriam Alvarado, Madhuvanti M. Murphy, Cornelia Guell

**Affiliations:** Chronic Disease Research Centre, Tropical Medicine Research Institute, University of the West Indies, Bridgetown, Barbados; Faculty of Medical Sciences, University of the West Indies, Bridgetown, Barbados; MRC Epidemiology Unit and UKCRC Centre for Diet and Physical Activity Research, University of Cambridge, Cambridge, UK

**Keywords:** Obesity, Physical activity, Gender, Caribbean, Qualitative research, Participant observation

## Abstract

**Background:**

The proportion of obese women is nearly twice the proportion of obese men in Barbados, and physical inactivity may be a partial determinant. Using qualitative interviews and ‘semi-structured’ participant observation, the aim of this study was to identify modifiable barriers to physical activity and to explore the factors that facilitate physical activity amongst overweight and obese women in this low-resourced setting.

**Methods:**

Seventeen women aged 25 to 35 years with a BMI ≥25, purposefully sampled from a population-based cross-sectional study conducted in Barbados, were recruited in 2014 to participate in in-depth semi-structured interviews. Twelve of these women participated in one or more additional participant observation sessions in which the researcher joined and observed a routine activity chosen by the participant. More than 50 hours of participant observation data collection were accumulated and documented in field notes. Thematic content analysis was performed on transcribed interviews and field notes using the software Dedoose.

**Results:**

Social, structural and individual barriers to physical activity were identified. Social factors related to gender norms and expectations. Women tended to be active with their female friends rather than partners or male peers, and reported peer support but also alienation. Being active also competed with family responsibilities and expectations. Structural barriers included few opportunities for active commuting, limited indoor space for exercise in the home, and low perceived access to convenient and affordable exercise classes. Several successful strategies associated with sustained activity were observed, including walking and highly social, low-cost exercise groups. Individual barriers related to healthy living strategies included perceptions about chronic disease and viewing physical activity as a possible strategy for desired weight loss but less effective than dieting.

**Conclusions:**

It is important to understand why women face barriers to physical activity, particularly in low-resourced settings, and to investigate how this could be addressed. This study highlights the role that gender norms and health beliefs play in shaping experiences of physical activity. In addition, structural barriers reflect a mix of resource-scarce and resource-rich factors which are likely to be seen in a wide variety of developing contexts.

## Background

Non-communicable diseases such as ischemic heart disease, stroke, and diabetes have been on the rise in the Caribbean region from 1990–2010. According to the Global Burden of Disease 2010 (GBD 2010), the median % change in disease burden attributable to diabetes increased 47 % (30-70 %) during the same period [1, 2]. The burden of disease in the Caribbean is increasingly shifting from communicable to non-communicable, chronic diseases [3]. A recent systematic review of social determinants of diabetes risk factors and control found that women across the Caribbean region were more likely to have diabetes, be obese and less physically active than men [4].

Based on GBD 2010 results, having a BMI ≥ 25 is the single greatest risk factor associated with disease burden in Barbados, contributing to over 11 % of total disability-adjusted life years (DALYs) [5]. According to the Health of the Nation Study, a population-based cross-sectional study conducted from 2012–2013, 74.2 % (70.3-77.7 %) of women aged 25 and over are overweight or obese compared to 57.5 % (51.2-63.7 %) of men [6]. The proportion of women and men who are obese varies dramatically, with obesity prevalence “almost twice as high in women (43 %) than in men (23 %)” [6]. The Health of the Nation study also reports prevalence of central obesity, which is more strongly associated with diseases such as diabetes and cardiovascular conditions. The prevalence of central obesity among women aged 25 and over (61.7 %, 58.0-65.2 %) is three times higher than in men (19.8 %, 16.0 %-24.2 %)[6]. Taken together, these data suggest that a deeper understanding of the determinants of this high gender-specific obesity prevalence may be important in curbing a growing public health concern. Physical inactivity was ranked fifth in the GBD list of risk factors that contribute to disease burden in Barbados, above both alcohol use and tobacco [7]. Based on objectively measured physical activity levels reported as part of the Health of the Nation study, 90.5 % (83.3-94.7 %) of women aged 25–54 are physically inactive according to WHO minimum recommendations, compared to 58.9 % (48.4-68.7 %) of men aged 25-54[6]. Moderate amounts of physical activity have been shown to help maintain weight and prevent weight gain [8], and physical activity has been shown to have a protective effect on a wide range of non-communicable diseases [9,10]. There are many evaluations of physical activity interventions in the United States, Canada, Europe, Australia and New Zealand [11–14]. However, little is known about the barriers and facilitators of physical activity in low resourced countries in Latin America and the Caribbean [15–21]. There have been numerous studies on the barriers and facilitators to physical activity among Afro-Caribbean populations in the Diaspora [22–24] but research about physical activity in the Caribbean is limited [25–27].

This work contributes to filling the gap. Qualitative research can add insights into the nuanced or competing influences that women may face. The combination of semi-structured interviews with participant observation (participating in study subjects' activities), adds methodological rigor to qualitative inquiry of physical activity. Opportunities include comparing 'what people say with what they do', gathering contextual data (such as on their housing or neighborhood environment), and revisiting previous interview comments and conversations in repeat visits [28]. A better understanding of women’s experiences may contribute to the design of messages and interventions that are more likely to be effective at increasing physical activity levels. This work aims to identify some of the barriers to physical activity currently faced by overweight and obese women in Barbados, and explore factors that facilitate physical activity successfully among this group.

## Methods

The research design is a qualitative case study, based on the Grounded Theory principles outlined by Strauss and Corbin. As an explanatory study of an under-researched topic, setting and population group, the research (recruitment, semi-structured interviews, participant observation sessions, transcription and analysis) was conducted in an ongoing and iterative basis to carefully explore the emergent data for explanatory insights [29, 30]. Data collection occurred from January to August 2014.

### Participants

#### Sampling

Seventeen female participants were recruited into the study. The purposeful sample included women between 25 and 35 years of age, having measured a BMI greater than or equal to 25, and who were not pregnant [28]. The relatively young age and narrow age band were chosen to match the primary investigator’s age and it was agreed that a closeness in age between the researcher and women included in the study may positively influence the comfort and candidness of the interviews and participant observation. That said, the primary investigator was an outsider to the setting (perceived as white/mixed race, American and slim, compared to the participants who were all black, Barbadian and overweight) and this may have still influenced data collection in terms of the information women were comfortable sharing, the topics discussed and the level of trust developed between the women and the primary investigator. Meeting the participants several times over a period of time helped to establish rapport and address potential concerns of the participating women. The sample was limited to women with a BMI greater than or equal to 25 to focus on a homogenous targeted sample, while allowing for variety in experiences. This focus would allow insight into whether and in what ways there may be opportunities for physical activity interventions to address issues of overweight and obesity. Pregnant women were excluded from the sample because of the assumption that physical activity patterns and experiences may be quite different during pregnancy. Although data on physical activity levels were collected as part of the Health of the Nation study, these data were not available at the time of recruitment and therefore the activity levels of women in the sample were not considered for analysis.

#### Recruitment

Following Grounded Theory principles, participants were recruited, visited, and their data analyzed through an iterative process, and recruitment ended when the team agreed that analytical saturation has been reached. To reduce the time between the initial recruitment phone call and the first interview, recruitment was staggered and done in batches of 4–5 women at a time. Based on our study design, we intended to recruit up to 20 women until saturation had been reached; this small number of participants was chosen with the aim to visit them repeatedly for more in-depth exploration of their experiences. All but one participant had been part of the Health of the Nation Study between 2012 and 2013 and at the time had indicated that they were willing to be contacted for future recruitment. The Health of the Nation study is a population-based cross-sectional study to define the burden of diabetes, CVD and associated risk factors by socio-economic status in the Barbados adult population [6]. Based on our sampling criteria, 53 women were eligible to participate, but not all eligible women were contacted. Of 36 women called, 12 could not be contacted due to restricted incoming calls, changed phone numbers or failure to respond after three messages. Of the remaining 24 women who were successfully contacted, all women agreed to participate in the study with the exception of one woman who declined. One woman was pregnant and therefore ineligible to participate. Once saturation was reached at 17 participants, the remaining eligible participants were not interviewed.

#### Pilot

To pilot the semi-structured survey instrument, one woman who was an acquaintance of the primary investigator and with whom rapport had already been established, was recruited from outside the Health of the Nation sampling frame in an effort to gain candid feedback about the survey instrument The pilot phase consisted of testing the semi-structured survey instrument for clarity and timeliness with this woman, after which it was decided that no further pilot testing was necessary as the instrument was clear and not overly time-consuming. Since the instrument did not change after the pilot and the woman who participated in the pilot met all of the other study requirements, her data has been included in the study because it enriched the data collected.

#### Participant characteristics

The final 17 participants had an average BMI of 33 (range 25–51), and represented 9 of the 11 total parishes in Barbados. Ten participants had completed college or higher and 14 were employed. Twelve women had children, of which five had one child, six had two children, and one woman had three children. Eight women were married. All women were of Afro-Caribbean descent. Further characteristics of the study participants are shown in Table 1.

**Table 1:** Participant Characteristics (n = 17)

**General**	
Average Age	30 years (27–34)
Average BMI	33 (25–51)
Number Married	8
Number with Children	12
**Education**	
Secondary school completed	5
Technical/secretarial after secondary school	2
College/university completed	9
Post graduate degree	1
**Occupation**	
Routine/Manual	1
Intermediate	7
Professional	6
Not in employment	3

### Procedure

#### Interviews

Eligible participants were contacted by phone, and an initial meeting was set up at a location of their choosing (typically either at home or a public space in the city). During this meeting, the basic aims of the study were discussed, and written informed consent obtained. Then, a semi-structured interview was conducted. The initial questions were designed to build rapport, and focused on family and daily activities. These included questions such as “Think back to yesterday. Can you walk me through your day? What time did you wake up? What did you do before breakfast?” Subsequent questions focused on transportation, leisure time, opinion leaders, social support, and media exposure. This section of the interview included questions such as “How did you get to [previously mentioned location]? What are some other ways that you get around? Who, if anyone, do you turn to for an opinion the most? In what situations?” A subcomponent of the interview focused on women’s experiences with physical activity, and their attitudes and beliefs about activity and exercise. During this portion of the interview some questions included the following: “How would you define physical activity? What kinds of physical activity did you do in the last week? Think back to the last time you did [previously mentioned form of activity]. What do you like about it? When did you do it? Who did you do it with?” Open-ended questions were used, with follow-up probes and clarifications. Interviews varied from 20 minutes to 57 minutes in length but averaged 30 to 40 minutes, and were audio recorded.

*Participant observations:* Following the initial interview, plans were made with each participant for a subsequent unstructured meeting based on the activities discussed during the interview. Twelve of the seventeen women participated in this second stage. Five women did not participate due to lack of time or lack of interest. Of the women who did participate, two were met with on multiple occasions (3+ times), two were met with twice, and the remaining 8 were met with once each. Activities included attending a family picnic, attending church, going to work together, going to parties, golfing, swimming, and attending various dance classes, going to the gym, walking to a childcare center, and walking home. These activities were selected based on activities discussed during the initial interview, or at the suggestion of the women themselves. As a result, the activities considered during participant observation varied widely. This was considered a strength of the methods, since physical activity is not limited to traditional exercise activities, and we wanted to capture both activity that occurs in non-exercise contexts (such as walking with children during a family picnic or stocking grocery shelves at work), as well as attitudes and perceptions about activity that may arise in non-active contexts (such as church leaders announcing exercise groups at church, or a co-worker sharing gendered opinions about physical activity). Field visits varied from one hour to six hours. A total of more than 50 hours of participant observation data collection were accumulated, and all observations were recorded as field notes. The first author (MA), a qualitatively trained female researcher, conducted all data collection, supervised by the second author (MMM) and the senior author (CG), both experienced qualitative researchers.

Ethics approval was gained from the Institutional Review Board of the University of the West Indies, Cave Hill, and the Ministry of Health, Barbados.

### Analysis

All interviews were transcribed verbatim, field notes typed up from handwritten notes and all data analyzed using Dedoose software. Codes were developed inductively from transcripts and field notes. An interim analysis was conducted after meeting with the first 10 participants. Quotations were clustered around broader themes and these themes were merged or altered where appropriate. Data collection continued until it was felt that saturation had been reached. All analysis was primarily conducted by the first author (MA). Since the nature of this research was very embedded, particularly with the participant observation component, it was not advantageous or necessary to use multiple coders. However, to ensure rigor, analysis was continuously and discursively checked by the second author (MMM), a locally based experienced qualitative researcher, and the senior author (CG), and any agreement over themes reached by consensus. Additionally, a public health doctoral candidate with expertise in physical activity was consulted independently. Themes were categorized into barriers and facilitators to physical activity. Finally, all 17 participants were revisited for member-checking, and findings were discussed and recorded. These recordings were used to update and refine results.

In this study, we use the term “physical activity” to refer to any movement that requires energy expenditure, and use the term “exercise” as a sub-category of physical activity that refer to activity done specifically to maintain or improve physical fitness in a structured setting. We use the term “active living” to refer to physical activity that is incorporated into daily life including commuting, at work, at home, and recreationally.

## Results

Results are presented in terms of barriers and facilitators according to broad themes and supporting evidence.

### Barriers

Barriers are subdivided into three broad categories: social, structural, and individual.

#### Social: Gender norms limit opportunities for exercise

Physical activity behavior seemed to be very gender-specific in Barbados. Women rarely reported and were rarely seen by the researcher exercising with men. Of the 10 participants that described ever doing some kind of activity during their leisure time, 9 were either alone or with female-only groups. Of the 5 women observed engaging in activity, none of them involved male friends or partners, and only one was a co-ed exercise class. During follow-up visits all but one woman in the study agreed that men and women were not active together. In contrast, women reported discussing exercise with other women and joining all-female exercise groups.

Me and my girlfriend we talk about it [physical activity] practically every day because she goes to a different gym to me […] So she would say what you do at gym today and I would say this and she would say yesterday I almost get killed!

(Alexis, late twenties, no children)

Asked about who they last heard talking about physical activity, some women explained that they talked with female friends about being active regularly. For these women, peer encouragement was reported to be a strong motivator. However, surprisingly some women described pulling away from female friends and acquaintances because of a perception that “it’s not good to have too many female friends” (Diana, early thirties, 1 child). Some women described a pervasive culture of distrust, explaining that women were often not genuinely supportive of one another.

I guess in Barbados how to explain it […] it’s like I don’t know when you have girlfriends, a lot of girlfriends, for some reason it creates problems. That’s all I could explain it […]. I don’t know if it is a Bajan [Barbadian] thing or a woman thing […]. Like you would find some women don’t like to find other women getting further than them.

(Dawn, mid thirties, 1 child)

I don’t really keep that many friends. I don’t honestly when I was young I used to have lots of friends and stuff and then. It wasn’t worth it […]. Honestly nobody want to see you getting through […] so I ain’t going to keep that many girlfriends.

(Diana, early thirties, 1 child)

When probed during follow up visits, most women agreed that this dynamic was pervasive. One woman disagreed strongly. This widespread experience of distancing from female peers may have reduced the effect of peer encouragement for physical activity. Out of the 6 women who described avoiding female peers without being prompted, 5 reported no leisure-time activity. Several of these women agreed that they would be more likely to be active if they had a female friend who would invite them to an exercise class or on a walk.

Pervasive gender roles also limited women’s opportunities to exercise in terms of their social responsibility as mothers and often household heads. Of the 12 women with children in this study, 4 women were single mothers. Some women had support from their extended family, while others did not. Several women described child rearing responsibilities as one of the primary reasons why they were not active. One single mother who was able to make exercise a priority described this dynamic:

Some women don’t have help - the children have dads that don’t help or other family members so that’s why too. They would have to put the children first. But I have help, luckily. That’s why I decided to start doing something [going to the gym].

(Shakela, early thirties, 1 child)

Other women mentioned the importance of having support from family in child rearing. Several women emphasized how prevalent single parent families were in Barbados, particularly single mothers. While spending participant observation time with one single mom at her workplace, she and a male colleague debated why she was not able to make time to exercise. She explained that with two kids she did not have the time or resources to join a gym, but he insisted that kids were not to be blamed and that she needed to make sacrifices in order to address her obesity.

The exchange captured both social norms about a woman’s role as a sacrificial mother, and little recognition of the social responsibilities and pressures of single mothers. Of note, this man and woman had the same job and the same professional obligations; the man had a child that did not live with him.

#### Structural: Socio-economic factors limit opportunities for active living

Participants reported experiencing a variety of socio-economic constraints, which were also observed during interviews and participant observation sessions.

First, some houses were too small to allow for exercising at home. These homes were typically *chattel houses*, which are small wooden houses (200 square feet) that were originally used during the plantation period in Barbados[31]. Some women described being active at home and doing a range of activities from Zumba® DVDs to light weight lifting and floor exercises to walking on a treadmill. However, for women living in smaller chattel houses, being active at home was observed not to be a viable possibility. During follow up visits, these women confirmed that it was difficult to engage in exercises at home.

Second, there was a perceived lack of access to convenient and affordable group exercise opportunities. For example, one woman said that she struggled to be active, explaining:

I would like to find a gym that is cheap that I could get a personal trainer. Ain’t cheap! Man, tell me is 60 dollars a session! That is 60 dollars for one session one 45 minute session! Not even an hour.

(Melissa, late twenties, 2 children)

Most participants were not aware of more affordable exercise options. This low awareness might be explained by the high visibility – both physically and in social media – of more expensive gyms that were described to be more effective. A few women highlighted that they did not seek out more affordable options because they did not consider them equally effective alternatives.

Third, opportunities for active commuting were limited in Barbados. Women who lived and worked in Bridgetown (Barbados’ capital) or other major commerce areas were able to walk to nearby stores, childcare facilities, or work. However, women who lived elsewhere typically were too far from these establishments to walk. Instead, most women drove or were driven by their partners. A few women described occasionally utilizing public transit. One woman described the barriers to active commuting:

Sometimes the walk is be good you know exercise but if I have my car I wouldn’t walk at all only when I don’t have do I walk cause everything closer in town [Bridgetown] that ya could walk to instead of wasting the gas but as for out here [St. Philip]… the closest shop there … nah… now that is daytime no way! Ain’t walking. Too hot!

(Janelle, mid-twenties, no children)

During the participant observation sessions, two women were observed walking as part of their commute. Both lived in Bridgetown. In participant observation of a morning spent with Maria (late twenties, three children), she took her daughter to daycare by bus and walked back. She carried her two-year old daughter for a while, commenting on how heavy she was. We walked along some pretty quiet roads until we turned into the nursery. On the way back she explained that normally she would walk with headphones as this was motivating and fun. The walk home took 25–30 minutes.

Motivation was clearly as important as the physical opportunity to walk for Maria. During this walk, Maria described that her other friends would never walk like she does. She explained that her friends would not walk because they all had cars and driving was more convenient, or they were lazy.

Since so many women reported and were observed to have access to cars, it seems that the incentive for active commuting is low.

### Individual: Health is not a major motivation for physical activity

Interviews also explored broad notions of health and women were asked if health was a motivation for anything they did. Without being asked, every woman in this study reported knowing someone with diabetes. While describing their motivations for pursuing physical activity, some women explained that witnessing friends and family with long term chronic conditions motivated them to take control of their own health as much as possible. One woman summarized this, saying “it’s really time for me to put things in order – if I still get diabetes when I’m skinny well that’s just life but it wouldn’t be because it’s my fault” (Krystal, late twenties, no children).

However, an equal number of other women spontaneously explained during initial interviews that the high prevalence of diseases like diabetes had led to have a lack of perceived self-efficacy around preventing long-term health conditions. One woman commented:

I do a lot of things that jeopardize the health but to me, regardless if you do or don’t do, things still happen […] Sometimes you will see a person don’t smoke don’t drink is still see them develop well everybody got diabetes so yeah but to me it don’t matter what you do or don’t to bring it on. Things just happen…

(Janelle, mid-twenties, no children)

During follow-up discussions, 8 women agreed that some women do adopt this mentality. One woman explained that Bajans [Barbadians] were known “as being laid back people […] we don’t rush or anything like that” (Krystal, late twenties, no children). She went on to assert that Bajan culture was characterized by the following belief:

What comes by is what’s supposed to happen. We don’t see a lot of initiative or someone wanting to do something different. [For example] none of my friends wanted to exercise […] it never happened I could never find someone to do it [be active] with me.

(Krystal, late twenties, no children)

For this woman, there was a clear link between this laid-back mentality and lower levels of physical activity.

Moreover, during initial interviews, some women explained that nutrition was a substitute for being active. They focused on healthy nutrition when they could not or chose not to be active. When asked about this during follow up visits, many women agreed that nutrition was perceived as a substitute for physical activity, and a few women described it as a more effective strategy for weight loss than being physically active. One participant summarized both of these views, saying:

I lacking in that area of exercise I try to make it up in how I treat my body what I put inside my body for as I see all the girls walking every evening and they look no smaller! Year now they walking and all of them big so and they walking everyday! But they ain’t going nowhere. Because when you see them at lunch and you see what they eating they’re wasting time walking.

(Kerrie, mid thirties, two children)

The dual perceptions that healthy nutrition was a substitute for being physically active and that nutrition was more effective for achieving weight loss seemed to act as barriers to engaging in physical activity. One woman explained her view:

To me it’s like nutrition - It’s like the biggest part in terms of you losing weight in terms of self control discipline like… how it’s like everyone thinks exercising like the main thing but I think it’s like the way that we eat that’s like more important because I ain’t say like for myself like sometimes when I take certain foods I can see myself my body feels better you know so just take nutrition it’s like the main key think like discipline has to start with that first.

(Lisa, mid-twenties, one child)

Another woman commented that there was an “idea that for some people they can just cut out bread and lose weight – so they don’t have to exercise” (Chelsea, mid thirties, one child).

Almost every woman in the study identified weight loss as the primary motivation for being active. No one discussed weight maintenance as a possible benefit or motivation associated with physical activity. Only two women identified health or fitness as a primary motivation. When asked about this, one woman explained:

You go to the doctor they gone tell you to lose weight – they never tell you to get healthy you know! They actually tell you to lose weight. So […] your goal is to lose pounds, it’s not to get healthy.

(Chelsea, mid thirties, one child)

With weight loss as the primary goal, many women apparently saw healthy nutrition as a potential substitute for being physically active. Extreme weight loss practices, such as diet clinics and diet pills, were also brought up by a few women. For example, one woman talked about attending a free consultation at a diet clinic:

They say it’s like 1500 dollars […] You could lose up to 30 pounds once you stay strictly to their diet […] Yeah you got to weight the food on a scale

(Melissa, late twenties, 2 children)

Melissa seemed very excited about this diet clinic and spontaneously brought it up several times over multiple visits. Despite being very conscious of saving money and not spending much on herself, she was willing to make this a priority and commented that she was starting to save for it. Another woman discussed having tried diet pills in the past, explaining:

It was very expensive but it used to work but I find out whenever I take them my heart used to race a lot […] so I stopped taking them I never took anything since that […] all they [diet pills] do is destroy you as you can see the two old ladies that we lost the other day from taking them. So yes you get results you get small and stuff but then afterwards the tablets and stuff doing other things to your body.

(Rebecca, late twenties, two children)

While physical activity was seen as one way to achieve weight loss, many women reported seeing healthy nutrition, dieting, and even diet pills as possible alternatives to being active.

### Facilitators

Despite these barriers, a subset of women reported being active regularly, and a series of participant observations involved physical activity. Discussions during interviews and participant observations revealed a set of factors that seem to facilitate physical activity among these women. These facilitators were typically described while answering questions such as “What did you do yesterday?” and “What did you do last weekend?” During discussions of these activities, the following facilitators were spontaneously described by a subset of women.

#### Social: Support & pressure as a motivator to stay active

In sustained exercise classes, most people seemed to know each other and teasing was a big part of the social experience. One woman who went to the gym four times a week described her reasons for going:

Going to the gym the motivation is, well obviously it would be generally to lose weight, but going by the gym is relatively small so you know everybody that is there so it’s kind of a family type atmosphere. And the guy that operates it he is funny and interactive […laughs] It doesn’t seem that horrible… Initially! [Laughs]

(Alexis, late twenties, no children)

Guilt around missing classes and fear of missing out were both reported to be strong motivations for consistent attendance. On a small island, several women explained that it was very likely that one would run into someone from an exercise group on the street, making social pressure more tangible. As Alexis went on to summarize, “It’s Barbados. It’s so small somebody always knows somebody that knows somebody.” She went onto explain:

I probably would pass somebody from my gym somewhere on the streets [… It’s] motivational in the sense that if you don’t go […to the gym] and pass a girl that I haven’t seen in a while ‘hey why I don’t see you in the gym? What’s going on with you?’ and I guess guilt people into coming back. So yeah it’s motivation.

(Alexis, late twenties, no children)

All of the women who participated in group exercise classes described experiencing this dynamic. In addition, Whatsapp groups comprised of women in the same exercise class could make this social pressure and social support even stronger. Whatsapp is a mobile-based application that was widely used in Barbados and functions in a similar way to traditional text messaging services. A Whatsapp group is analogous to a group text, and allows members who were invited into the group to exchange messages in a common forum or thread. One woman described a Whatsapp group amongst her Zumba® classmates:

I get support from friends because umm… if you going we have a chat so if you are going might ask ‘You going to Zumba® tonight?’ Even if you thinking about going and you saying well I got this here to do[…] them’s encourage you to go like you’s feel alright they going so I’m going.

(Rebecca, late twenties, two children)

There seemed to be a camaraderie between classmates and trainers as well. One woman recounted missing a day at her gym after Kadooment, a national carnival-like festival. Her trainer playfully messaged her saying “What, are you still drunk?” She quickly promised to be back the following day.

#### Structural: Access to opportunities for active living do exist

Many women spontaneously reported being highly aware of the ways they engaged in active living outside of traditional exercise. Some women described a variety of strategies, including walking, dancing and active jobs as ways that they were active regularly. While not all women perceived access to opportunities for physical activity, based on observations some women did encounter structures (relatively safe neighborhoods, affordable exercises classes) that enabled physical activity.

While opportunities for active commuting seemed limited, interviews and participant observation revealed that most women could walk in their neighborhoods or easily access spaces to walk. Walking was regarded as flexible and free. For one woman, walking was the most convenient form of activity because she could squeeze it into an otherwise busy day. She described driving her son to track practice, and explained:

Instead of sitting inside the car for the two to three hours I decide just to walk. It’s calming the headphones in I listening to on the radio and… get time to reflect on the day and you know time to wind down […] it’s like good for your mental let the day go and relax and reflect.

(Chelsea, mid thirties, one child)

Another woman described two different kinds of walks:

Those walks are not exercise walks those walks are walks of leisure ahhhh – sigh –breathing you know like that’s what my walks are. Walks to clear your mind free yourself – exercise walking it would be like early in the morning but I ain’t do that for a long time.

(Melissa, late twenties, 2 children)

For the women who did describe walking in their leisure time, two out of the three did it primarily to relax. For these women, exercise was not the main motivation behind these kinds of walks. Instead, reducing stress and relaxation were reported as being important motivators for sustained walking.

Other women discussed dancing when asked about physical activity, and several additional women were observed dancing during the participant observation sessions as well. Women discussed many available spaces for dancing, and dancing was often mentioned as a preferred form of recreation. Again, exercise was not the main motivation, rather women saw this kind of dancing as fun and social. However, the women who talked about dancing were aware that it was highly active. When asked about physical activity, one woman responded:

Dancing. Tha’s the only physical activity - I don’t do the exercises […] I dance so much I feel like I burn a lot of calories. Ya’s be sweating! I sweating down boy!

(Janelle, mid-twenties, no children)

While some women reported active living as recreation and entertainment, others described how they either had active jobs or found ways to incorporate physical activity into their workdays. For example, Melissa was very aware of the activity involved in her job, and viewed it as a major benefit of the position. She described her dissatisfaction with various previous jobs:

I worked as […] a cashier at a supermarket until 2009 and you know a cashier sits down ain’t much activity in that and then in 2009 to 2011 I did secretarial work – so that’s even worse! Cause […] when you’re sitting down and working all day sitting down Facebook – you know after you ya know finish your work – but then […] I got this new job that I totally love cause since I really can’t get the exercise that I want to put in, I think it give me a little moderate […] up and down.

(Melissa, late twenties, two children)

Women with more sedentary jobs described seeking out opportunities to be active as well. For example, Dawn, an accountant, explained how she made time to walk throughout her day:

Like if I don’t have anything to do at my desk I’s just walk about and shout people that kinda stuff […] my office is split so the other part of my office is far if I have to get something signed I have to walk to get it signed. Like… about seven minutes.

(Dawn, mid-thirties, one child)

Rebecca, who worked at an office, described how she was proactive about walking also:

I push even when I’m at work I carry along my flat shoes and I push myself across the office I like if anybody want anything I offer to go for it just to get to and fro.

(Rebecca, late twenties, two children)

Some women also found affordable and convenient group exercise options. For example, one woman attended a Zumba® class that was taught in her neighborhood and cost BB$3 [US$1.50] for a 90-minute session. This was quite an exceptional community-led effort, but demonstrates that it was possible to create and sustain affordable and accessible group exercise classes. She explained:

If I had a reasonable gym close to me, that probably on evenings before I go I could go there and then come home but the gyms near my side are like 300 and something [BB] dollars a month 400 and something [BB] dollars a month […] and this actual Zumba® you go to we do it 3 [BB] dollars a day which is 9 [BB] dollars basically a week for three times which to be honest the work out we do is basically we could just call it a week.

(Rebecca, late twenties, two children)

Another woman described exercising at a backyard gym in Bridgetown, which cost BB$10 [US$5] per week.

Reflecting the range in socio-economic circumstance in the participants, there was a wide range in what women considered affordable. Another woman attended a different Zumba® class for BB$18/session [US$ 9/session], and another participant consistently paid BB$180 [US$90] for six pole dancing classes. Although cost was both a real and perceived barrier, there were some options that were more affordable for women across the socioeconomic range.

### Individual: Positive experiences act as motivators

Even when health and fitness were not reported as the primary motivations for initiating physical activity, women who joined exercise groups that were fun and that they attended consistently described seeing real health benefits. Women talked about experiencing less shortness of breath, decreased knee and ankle pain and increased cardio endurance. One participant described being motivated by an improvement in an old injury:

When I was younger I twisted my ankle so I always used to have to wear ankle braces but I found that once I got heavier it would tend to roll out more and I remember [… ] anytime I do anything like walking or hiking I’m going to have to wear this brace […] Now that I’m working out sometimes I’m like oh crap I don’t have the brace –! [Laughs] Because I haven’t rolled it for that long, maybe like two years. It’s a huge difference.

(Krystal, late twenties, no children)

While these health-related benefits were not part of her initial motivation, this participant went on to explain that seeing this change has motivated her to *continue* being active.

## Discussion

This qualitative study with Afro-Caribbean women explored barriers and potential motivators to physical activity. First, social gender norms seemed to restrict how and with whom women chose to be active. Most women reported only exercising with other women, lacking a significant group of female peers for encouragement, and facing time and financial constraints related to child-rearing responsibilities, particularly as single mothers. Second, women faced a variety of barriers related to socioeconomic factors. Cramped housing limited indoor exercise opportunities, and organized exercise classes and gyms were generally perceived to be very expensive by women with limited disposable income. Interestingly, despite varied levels of income most women had access to a car, so incentives for active commuting were limited. Finally, some women perceived chronic disease as random and inevitable, and were not convinced of the link between being active and long-term health. Many women highlighted the benefit of weight-loss over other health concerns. Some women reported focusing their efforts on healthy nutrition as a substitute for physical activity.

It was not assumed at the outset of the study that the participants, who were all overweight and obese, were physically inactive. Some women indeed discussed and were observed to be active regularly. For these women, several factors seemed to encourage consistent activity. First, social support and pressure, which were perceived to be heightened on a small island, provided additional motivation for remaining consistently active. Second, some women discussed walking for relaxation, and walking was observed to be an accessible form of physical activity for most women. A subset of women as also able to find exercise groups that were affordable, across a range of socioeconomic levels. Third, while health was rarely discussed as a primary motivation for being active, women reported seeing distinct health benefits to regularly engaging in activity, and positive experiences, for example improved fitness, were additional motivation to continue.

The study complements findings from qualitative research on women’s perceptions of and barriers around physical activity predominantly set in North-American and European settings, but also highlights complexities that may be more pronounced in lower resourced settings such as the Caribbean in which qualitative physical activity studies are still missing [32, 33].

Several studies reported weight loss as a strong motivator for engaging in physical activity, greater or equal to the motivation of health benefits [34, 35]. However, dieting was preferred by some of our participants over physical activity as an effective weight loss strategy. Their experience mirrors scientific evidence that suggests that a high amount of physical activity seems to be required for effective weight loss [36–39]. One consensus panel of physical activity experts suggested that “prevention of weight regain in formerly obese individuals requires 60–90 minutes of moderate intensity activity”[37]. Another intervention study found significant long term weight loss was achieved in a group that was instructed to walk around 75 minutes per day as compared to the standard recommendation of 30 minutes per day [38]. In face of competing priorities in these women’s lives, perceived quicker weight loss strategies trump physical activity.

A more sustainable motivator than weight loss ambitions seems to be social support, in particular the role of female friends as exercise partners. Gender differences in social support are described elsewhere, with girls/women appreciating warmth, encouragement and social exchange from their peers while boys/men highlight exchange of skills, status and information [40, 41]. In our study, women echoed the need for social support from their peers. A subset of women, however, described isolation from their female peers in adulthood as a significant barrier to sustained physical activity. Programs that promote community-building and focus on developing and maintaining relationships amongst women (and the broader community) may be helpful at reducing the social isolation some women described, and may ultimately support active living. In particular, low-cost community-based exercise classes or walking groups have the potential to address some of the barriers to being active while amplifying some of the facilitators identified in this study, such as social support. While a low-cost Zumba® class was identified, it was also discussed as being quite exceptional. Finding ways to support the development of additional low-cost dancing classes throughout the island may be one way to increase access to affordable, women-friendly exercise opportunities. Social responsibilities were also reported as strong barriers to physical activity in women elsewhere [42]. Time and resource constraints related to child care, work and household duties may be particularly linked to structural disadvantage [42]. In the setting of this study, single mothers, who were household heads, particularly faced this double burden and find it difficult to incorporate physical activity into their daily lives. Barbados – and the Caribbean region more widely – has one of the highest rates of single-mother families in the world [43, 44].

Of course single motherhood and the double burden of household and work responsibilities are common in other settings, in particular in lower income populations. Research suggests that active living – the combination of physical activity derived from travel, work, household and leisure activities – is therefore a relevant and applicable concept for disadvantaged women who have less time for structured exercise but may nonetheless be physically active [42]. A case in point is active travel, which is more commonly practiced amongst disadvantaged women [42, 45]. In the Barbadian setting, however, we found in our sample that car ownership or at least access to car use was widespread, despite the relatively low disposable income in many of our participants. Although the physical environment could be made to facilitate activity more – in particular infrastructural improvements to encourage active travel, there are currently safe and affordable options available, such as walking locally or using the many public parks and beaches that are generally safe spaces. This is in contrast to findings from other studies primarily based in high resourced countries – but often deprived and therefore unsafe – urban centers [42, 46, 47].

### Strengths and limitations

Including a participant observation component greatly enriched the study’s findings. For example, through participant observation a variety of locally-led physical activity initiatives were identified. In addition, spending time with women in a variety of contexts allowed for insight into social and structural barriers that were not reported but observed consistently and confirmed during follow-up visits.

This study also faced several limitations. In-depth participant observations were made with 12 of the 17 women in this study. Women who did not participate may have been systematically different – busier or less active, for example in ways that may be related to the study objectives. The study focused specifically on women, but an investigation of male perceptions, attitudes and behaviors may help to highlight gender-specific determinants of differing obesity rates. In addition, the sampling frame was limited. The first limitation was to only include women ages 25–35. However, younger and older women may have very different experiences and motivations. Teenage girls typically have very low physical activity levels, but we did not have access to this age group based on the age range of the Health of the Nation study[6]. Equally, older women with different responsibilities may face different barriers and hold different perception regarding healthy living. However we excluded older women due to the primary investigator’s age and our assumption that sharing a proximity in age with the women in this study would affect the comfort and candidness of interviews. The sample was also limited to overweight and obese women, to investigate the current relationship between these women and physical activity and possibly inform future physical activity interventions targeting this group.

## Conclusions

The findings of this study suggest that gender-specific social norms and structural circumstance are important determinants of female physical activity in overweight and obese women. Future public health interventions could focus on fostering peer support, either in terms of exercise classes, buddy-schemes or more unstructured walking groups. Affordable and accessible group exercise classes, for example locally-led low-cost and ‘female-friendly’ Zumba® classes, are feasible within a Caribbean context and successful models have the potential to be replicated, particularly if they were subsidized to make them more accessible. This study also supports a general emphasis on active living as a population-based strategy towards increased physical activity that communicates to women that active lifestyles can be achieved while working, and through recreation such as dancing.
